# Informing urban climate planning with high resolution data: the Hestia fossil fuel CO_2_ emissions for Baltimore, Maryland

**DOI:** 10.1186/s13021-020-00157-0

**Published:** 2020-10-14

**Authors:** Geoffrey S. Roest, K. R. Gurney, S. M. Miller, J. Liang

**Affiliations:** 1 School of Informatics, Computing, and Cyber Systems, Northern Arizona University, Flagstaff, AZ USA; 2 Department of Environmental Health and Engineering, Johns Hopkins University, Baltimore, MD USA; 3 School of Life Sciences, Arizona State University, Tempe, AZ USA; 4 Present Address: ESRI, Redlands, USA

**Keywords:** Fossil fuel, Carbon dioxide, Emissions, Hestia, Baltimore

## Abstract

**Background:**

Cities contribute more than 70% of global anthropogenic carbon dioxide (CO_2_) emissions and are leading the effort to reduce greenhouse gas (GHG) emissions through sustainable planning and development. However, urban greenhouse gas mitigation often relies on self-reported emissions estimates that may be incomplete and unverifiable via atmospheric monitoring of GHGs. We present the Hestia Scope 1 fossil fuel CO_2_ (FFCO_2_) emissions for the city of Baltimore, Maryland—a gridded annual and hourly emissions data product for 2010 through 2015 (Hestia-Baltimore v1.6). We also compare the Hestia-Baltimore emissions to overlapping Scope 1 FFCO_2_ emissions in Baltimore’s self-reported inventory for 2014.

**Results:**

The Hestia-Baltimore emissions in 2014 totaled 1487.3 kt C (95% confidence interval of 1158.9–1944.9 kt C), with the largest emissions coming from onroad (34.2% of total city emissions), commercial (19.9%), residential (19.0%), and industrial (11.8%) sectors. Scope 1 electricity production and marine shipping were each generally less than 10% of the city’s total emissions. Baltimore’s self-reported Scope 1 FFCO_2_ emissions included onroad, natural gas consumption in buildings, and some electricity generating facilities within city limits. The self-reported Scope 1 FFCO_2_ total of 1182.6 kt C was similar to the sum of matching emission sectors and fuels in Hestia-Baltimore v1.6. However, 20.5% of Hestia-Baltimore’s emissions were in sectors and fuels that were not included in the self-reported inventory. Petroleum use in buildings were omitted and all Scope 1 emissions from industrial point sources, marine shipping, nonroad vehicles, rail, and aircraft were categorically excluded.

**Conclusions:**

The omission of petroleum combustion in buildings and categorical exclusions of several sectors resulted in an underestimate of total Scope 1 FFCO_2_ emissions in Baltimore’s self-reported inventory. Accurate Scope 1 FFCO_2_ emissions, along with Scope 2 and 3 emissions, are needed to inform effective urban policymaking for system-wide GHG mitigation. We emphasize the need for comprehensive Scope 1 emissions estimates for emissions verification and measuring progress towards Scope 1 GHG mitigation goals using atmospheric monitoring.

## Background

Cities represent more than half of the global population, 67–76% of global energy use, and 71–76% of the carbon dioxide (CO_2_) emissions associated with final energy use [1], highlighting the need for sustainable urban development. Cities around the globe are responding to the inertia of nation-state scale climate change mitigation efforts. Organizations such as the C40 cities program (https://www.c40.org/), ICLEI—Local Governments for Sustainability (https://www.iclei.org/), and the Global Covenant of Mayors for Climate & Energy (GCMCE, https://www.globalcovenantofmayors.org/) have participating member cities across the world (94 cities in C40, more than 1750 member governments in ICLEI, and more than 10,000 cities in GCMCE). The US epitomizes this trend; more than 82% of the population lives in urban areas as of 2018, a number that is expected to rise to 89% by 2050 [2], and groups like Climate Mayors (http://climatemayors.org/) have rapidly grown following the announcement that the US federal government would be leaving the Paris Agreement.

Many cities have been setting sustainability goals, including greenhouse gas (GHG) emissions reductions, and some cities have even set emissions goals more stringent than the Paris Agreement, which seeks to cap global warming at 1.5 °C above preindustrial levels [3]. In order to meet their stated goals, cities typically start with an inventory of their baseline emissions following one of a few established protocols and inventory tools that accompany them. These protocols/tools are often developed by non-governmental organizations to assist cities with the baseline inventory process and reflect the entire-city scale within broad sectoral categories (We refer to these inventories as ‘self-reported inventories’—SRIs). While these tools are relatively simple and accessible, limitations in data, city staff time, and budget constraints mean that the methods, baseline year, estimation quality and comprehensiveness, vary from city to city. Furthermore, these SRIs often lack granular spatiotemporal patterns needed to identify high-emitting activities or infrastructure that can be effectively targeted in climate mitigation policy [4]. For example, cities may focus on improving traffic flow along the highest-emitting road segments to reduce CO_2_ emissions from vehicle congestion [5, 6]. Finally, they are often a mixture of the traditional emission scopes—Scope 1 (all emissions within a geographical boundary), 2 (emissions driven by the consumption of electricity), and 3 (emissions driven by the consumption of materials, goods, and services) [7, 8]. This mixture may accommodate city-specific emissions policies or monitoring requirements, but it limits the ability of the SRIs to be compared to atmospheric monitoring, the only current means to independently evaluate emissions and provide constructive feedback on the uncertainties in Scope 1 emissions to city planners [9, 10].

Although most SRIs do not resolve granular patterns in FFCO_2_, there have been several recent efforts to do so at different spatial scales, often as a component of research into biogeochemical cycling and climate change. Multiple national- and subnational-scale emissions estimates of Scope 1 CO_2_ emissions from fossil fuel combustion (FFCO_2_), the dominant component of anthropogenic GHG emissions, have been developed in the US at fine spatiotemporal resolution. The Anthropogenic Carbon Emissions System (ACES) dataset [11, 12] provides FFCO_2_ emissions for the north-eastern US at an hourly, 1 km resolution and has been used in an urban CO_2_ inverse modeling study in Boston [13]. The Database of Road Transportation Emissions (DARTE), which is the onroad component of ACES, has been expanded to the entire US domain [14]. Meanwhile, the Vulcan product provides hourly, 1 km FFCO_2_ emissions for the contiguous US and Alaska for 2010 to 2015 [15, 16]. Vulcan has been tested against atmospheric ^14^CO_2_, a particularly good tracer for FFCO_2_ (as opposed to CO_2_) and was found to be within 1.4% of the estimated national total derived from atmospheric observations [17]. Both ACES and Vulcan leverage data from the US Environmental Protection Agency’s National Emissions Inventory (USEPA NEI) for 2011 [18], hereafter referred to as the ‘2011 NEI’, among other data sources. The USEPA NEI is an inventory of emissions of ‘criteria air pollutants’, ‘hazardous air pollutants’, and other important pollutant precursors that is produced once every 3 years. In both ACES and Vulcan, carbon monoxide (CO) emissions from combustion sources in the 2011 NEI were converted to FFCO_2_ using ratios of emission factors. Output from Vulcan at the county scale is used as input data for Hestia—a city-specific, highly granular FFCO_2_ emissions product for urban domains, wherein emissions from Vulcan are redistributed in space and time using local data (e.g. building footprints and local traffic data) [19, 20]. Hestia was first developed for Indianapolis, spawning the INFLUX project [9, 10, 21–23], which has tested the accuracy of a GHG measurement system that integrates bottom-up emissions modelling, atmospheric measurements, and inverse modelling. Hestia has since been developed for Salt Lake City [24], the Los Angeles Megacity [25], and Baltimore [26]. This paper summarizes the methods and data sources for the Hestia-Baltimore product, which is available at an hourly resolution on a 200 m grid [27], and provides an overview of spatiotemporal patterns within the city for various sectors.

The present study specifically focuses on Baltimore, Maryland, and there are several existing SRIs in support of local emissions goals. The Baltimore Office of Sustainability (https://www.baltimoresustainability.org/) periodically releases sustainability plans for the city. The 2009 sustainability plan established a goal to reduce Baltimore’s GHG reductions by 15% by 2015, over baseline emissions of 7579,144 t (Mg) CO_2_eq in 2010. Building energy use accounted for 79% of those emissions. The city then published a Climate Action Plan in 2013 [28], which redefined the reduction target to 15% below 2010 levels by 2020. The plan focuses on energy savings and supply, land use, transportation, and growth.

We present a comparison of the Hestia-Baltimore Scope 1 emissions output with the city’s 2014 SRI (2020 email from L. McNeilly to S. M. Miller and G. S. Roest, unreferenced, see notes) and highlight differences in scoping, assumptions about source sectors, and data sources. The aim is to isolate the critical assumptions or data choices that lead to estimation differences and propose recommendations for generating urban Scope 1 emissions estimation that can satisfy the requirements of atmospheric verification and meet city needs related to specific mitigation policy approaches.

## Results

**Fig. 1:**
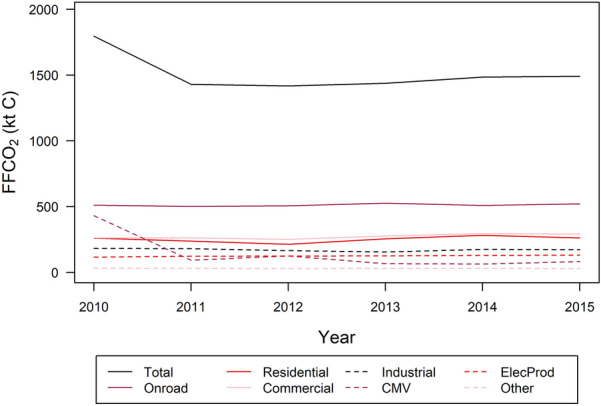
Annual timeline of emissions by sector in Baltimore. The ‘other’ category contains nonroad, rail, and aircraft emissions. Nonroad emissions dominate these three sectors. The drop in annual total emissions from 2010 to 2011 and beyond is driven by a decrease in CMV emissions, which reflects a decrease in fuel sales in EIA data after 2010

The Hestia-Baltimore v1.6 Scope 1 FFCO_2_ emissions are reported for the 2010 to 2015 (Table 1), with a focus on 2014 (Fig. 1). Emissions totaled 1487.3 kt C (Mg C) in 2014, with a 95% confidence interval (CI) of 1158.9 to 1944.9 kt C. The central estimate of annual emissions ranged from a peak of 1798.3 kt C in 2010 to a minimum of 1420.0 kt C in 2012. After 2012, the total emission increased each year through 2015. The peak in emissions in 2010 was driven largely by the commercial marine vessel (CMV) sector. The largest emitting sectors in each year except for 2010 were onroad, commercial, residential, and industrial, representing 34.2%, 19.9%, 19.0%, and 11.8% of the total emissions in 2014. In 2010, however, CMV emissions were the second largest emitting sector behind onroad. The electricity production and nonroad sectors each accounted for less than 10% of the total emissions in each year, while rail and aircraft emissions accounted for less than 1%. Table 2 shows the emissions categories organized by sector and scope that were included in Hestia-Baltimore and the City’s 2014 SRI (2020 email from L. McNeilly to S. M. Miller and G. S. Roest, unreferenced, see notes). Hestia-Baltimore includes only Scope 1 FFCO_2_ emissions while the SRI includes most Scope 1 categories, Scope 2 emissions in buildings and associated with electrified railways, and Scope 3 emissions associated with waste. Our comparison is restricted to only FFCO_2_ from the common Scope 1 categories.

**Table 1:** Hestia-Baltimore annual sectoral emissions and the percentages of the total annual emissions in Baltimore

Source ↓	Year →	2010	2011	2012	2013	2014	2015
Residential	kt C	260.7	238.7	214.4	255.4	282.5	262.3
	%	14.5	16.7	15.1	17.7	19.0	17.6
Commercial	kt C	262.8	262.6	252.1	277.8	296.3	292.5
	%	14.6	18.3	17.8	19.3	19.9	19.6
Industrial	kt C	182.3	180.6	165.6	155.3	175.3	172.8
	%	10.1	12.6	11.7	10.8	11.8	11.6
Electricity production	kt C	115.8	122.3	124.7	126.3	129.1	131.5
	%	6.4	8.5	8.8	8.8	8.7	8.8
Onroad	kt C	511.7	501.1	506.1	526.3	508.9	521.3
	%	28.5	35.0	35.6	36.6	34.2	34.9
Nonroad	kt C	28.6	27.6	27.5	27.7	27.2	27.9
	%	1.6	1.9	1.9	1.9	1.8	1.9
Aircraft	kt C	1.6	1.5	1.2	1.1	0.6	0.8
	%	0.1	0.1	0.1	0.1	0.0	0.1
Rail	kt C	3.6	3.8	2.4	3.7	4.0	1.7
	%	0.2	0.3	0.2	0.3	0.3	0.1
CMV	kt C	431.3	93.2	126.0	66.3	63.4	83.0
	%	24.0	6.5	8.9	4.6	4.3	5.6
Total	kt C	1798.3	1431.5	1420.0	1439.8	1487.3	1493.9
	%	100.0	100.0	100.0	100.0	100.0	100.0

Emissions are reported for the central emissions scenario [16]. Sums of individual sectors may not match totals due to rounding. The units of FFCO_2_ emissions are kt C (Mg C)

**Table 2:** A comparison of 2014 GHG emissions in Hestia-Baltimore and the 2014 SRI

Sector	Fuel	Hestia-Baltimore Scope 1 (95% CI)	SRI Scope 1	SRI Scope 2	SRI Scope 3	SRI Total
Residential^a^	Natural gas	244.6 (168.9–362.2)	204.4	–	–	439.6
	Petroleum	37.8 (26.4–55.5)	–	–	–	
	Electricity	NA	–	235.2	–	
Commercial^a^	Natural gas	276.0 (195.4–400.3)	130.4	–	–	459.7
	Petroleum	20.2 (14.5–29.1)	–	–	–	
	Electricity	NA	–	329.2	–	
Industrial	Natural gas	28.8 (22.1–38.8)	155.7	–	–	458.4
	Petroleum	146.5 (110.0–201.9)	–	–	–	
	Electricity	NA	–	302.4	–	
Onroad^b^		508.9 (436.7–581.2)	593.7	–	–	593.7
Electricity generation		129.1 (112.3–145.9)	2186.7^c^	–	–	2186.7
Rail^d^	Fossil fuels	4.0 (3.3–5.2)	–	–	–	9.5
	Electricity	NA	–	9.5	–	
Nonroad		27.2 (26.1–28.2)	–	–	–	–
Aircraft		0.6 (0.5–0.9)	–	–	–	–
CMV		63.4 (42.8–95.8)	–	–	–	–
Solid waste generated in city		–	6.1	–	–	6.1
Incinerated waste generated in city		–	–	–	0.1	0.1
Wastewater generated in city		–	6.4	–	–	6.4
Total^e^		1487.3 (1158.9–1944.9)	1096.8	876.4	0.1	1973.2

Emissions of FFCO_2_ for Hestia-Baltimore and CO_2_e for the SRI are reported in kilotonnes of carbon (kt C). The 95% confidence intervals for Hestia-Baltimore emissions are provided in parentheses. The SRI includes emissions of methane and nitrous oxide as well. “NA” denotes a Scope 2 emissions category that was never intended to be quantified in Hestia-Baltimore Scope 1 emissions

^a^Hestia-Baltimore includes negligible emissions from coal consumption in the residential and commercial sectors

^b^Onroad emissions in Hestia were disaggregated by fuel type (gasoline and diesel) while the onroad emissions in the SRI were disaggregated by vehicle class. The sum of onroad emissions from all fuels and vehicle classes is presented in the table

^c^The SRI includes emissions from electricity generation from facilities that are not within the geographical city boundary in the Scope 1 total, though these are not technically Scope 1 emissions for the City. The reported Scope 1 FFCO_2_ emissions for only the facilities that are within the city boundary total 103.0 kt C

^d^Hestia-Baltimore only contains rail emissions from fossil fuel burned by prime movers on trains and emissions from railyards while the SRI only contains Scope 2 emissions from electrified railways

^e^The totals in reported in the SRI do not include emissions from energy generation supplied to the grid because these emissions would be double counted with Scope 2 emission. In this manuscript, Scope 1 emissions from the SRI are quantified and included in the comparison with Hestia-Baltimore (Tables 5 and 6)

### Building sectors

#### Residential

**Fig. 2:**
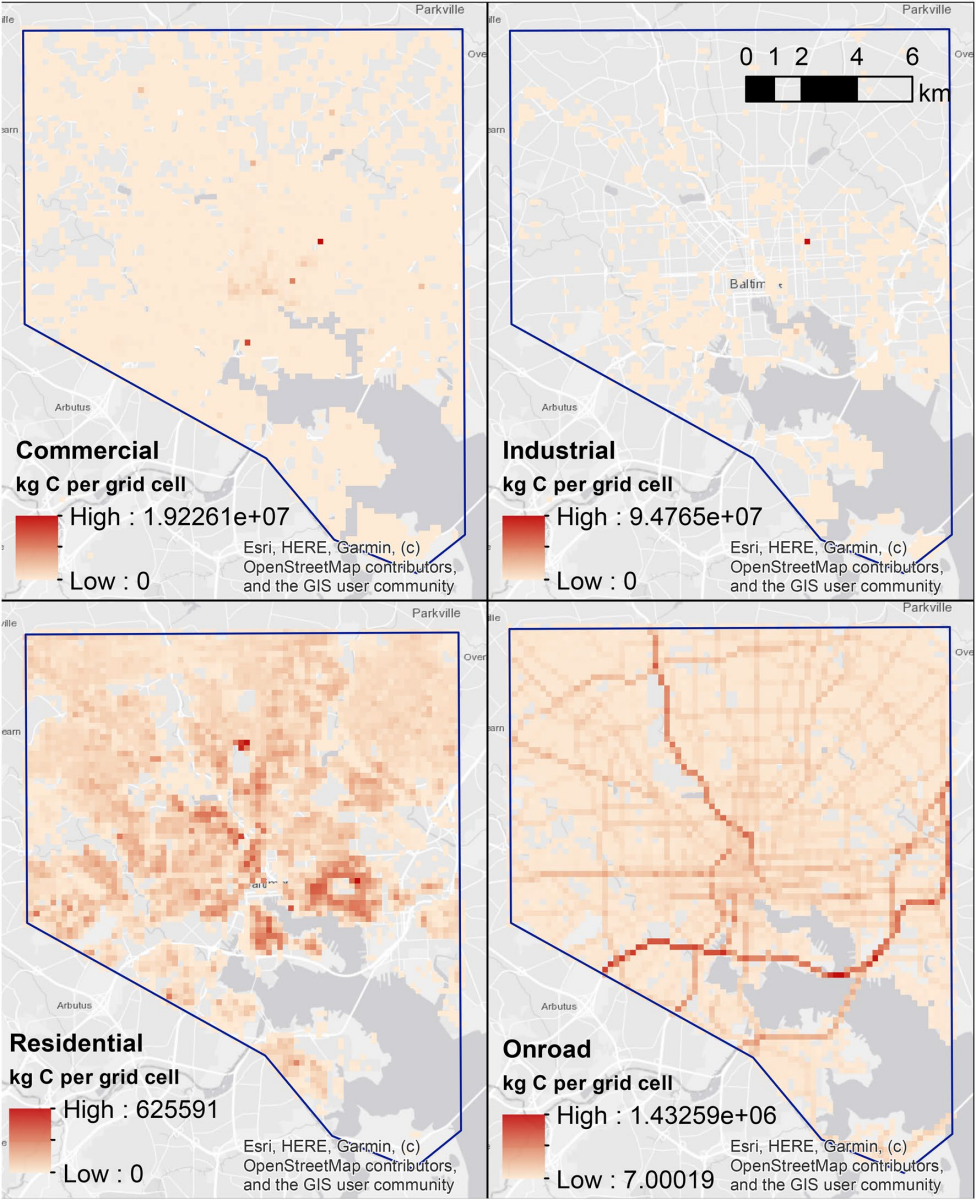
Gridded emissions for the Hestia-Baltimore for 2014. The sectors shown are commercial (top left), industrial (top right), residential (bottom left), and onroad (bottom right). This map was made in ArcGIS v10.6.1 using the Light Gray Canvas basemap (Esri, HERE, Garmin, (c) OpenStreetMap contributors, and the GIS user community)

**Fig. 3:**
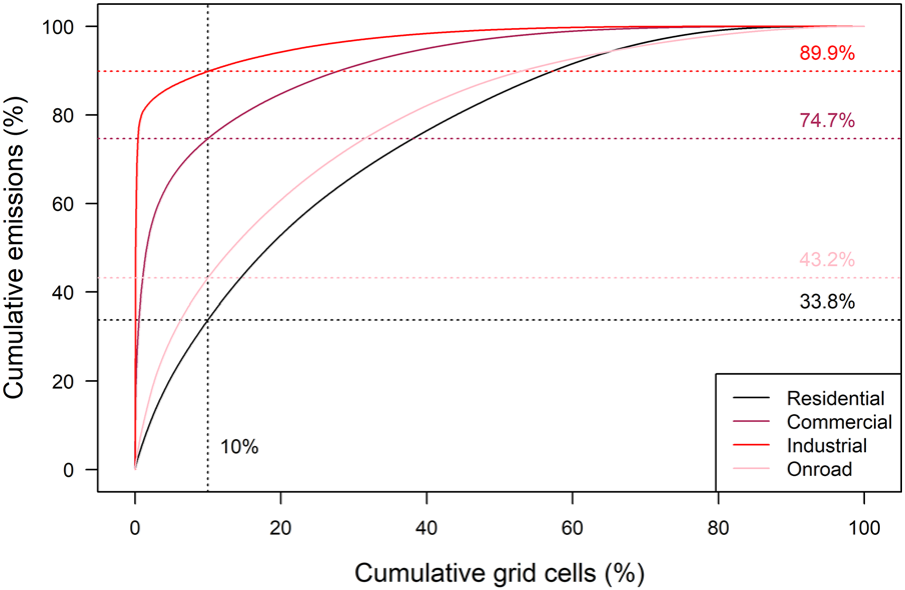
Cumulative emissions in grid cells for the residential, commercial, industrial, and onroad sectors for 2014. The intersection of the vertical dotted line and the sectoral emissions represents the share of emissions that are contained within the top 10% of non-zero emitting grid cells. The top 10% of grid cells contain 89.9% of industrial emissions, 74.7% of commercial emissions, 43.2% of onroad emissions, and 33.8% of residential emissions

The residential sector emissions of 282.5 (195.3–417.7) kt C represented 19.0% of the total city emissions in 2014. Natural gas consumption was the source of 86.6% of those emissions while petroleum fuels (liquid fossil fuels, e.g. distillate fuel oil and kerosene) contributed nearly all of the rest. Residential coal consumption was negligible. Figure 2 shows the 200 m gridded annual residential FFCO_2_ emissions for 2014 and Fig. 3 shows the cumulative fraction of emissions in all emitting grid cells. The top 10% of emitting grid cells were responsible for 33.8% of residential FFCO_2_ in Baltimore, while the top 50% of grid cells accounted for 84.8% of residential emissions.

Residential FFCO_2_ emissions were only reported for natural gas consumption in Baltimore’s SRI (Table 2). The residential natural gas emissions in 2014 were estimated to be 244.6 (168.9–362.2) kt C in Hestia-Baltimore and 204.2 kt C in the SRI. The SRI emissions were within the 95% CI of Hestia-Baltimore emissions with a relative mean difference of 18.0% for the central emission estimate. The 2014 SRI documentation states that residential GHG emissions in Baltimore were estimated using Baltimore Gas and Electric (BGE) utility data on natural gas energy consumption for single- and multi-family residences while petroleum consumption was not estimated, likely due to the fact that home heating oil is often purchased through private transactions instead of from a centralized utility. The exclusion of petroleum is an important data gap in the SRI as 13.4% of residential FFCO_2_ was associated with petroleum fuels in 2014. The 2011 NEI data, which serves as input to Vulcan and subsequently Hestia (see “Method**s**” section), includes estimates of nonpoint criteria pollutant emissions associated with petroleum combustion—hence, the data used by Maryland to report emissions to the NEI, if available, may be useful for developing city-scale GHG emissions estimates.

#### Commercial

The commercial sector emissions total of 296.3 (209.9–429.4) kt C in 2014 (19.9% of the annual total) made this sector the second largest FFCO_2_ source in the city, with 77.3% from nonpoint commercial buildings and the remaining 22.7% from commercial point sources. Figure 2 shows the prominence of commercial point sources within high emitting grid cells. The top 10% of emitting grid cells were contained 74.7% of emissions (Fig. 3). Natural gas use was associated with 93.2% of commercial FFCO_2_ emissions with petroleum accounting for 6.8%. Less than 0.1% of commercial FFCO_2_ was associated with coal.

Commercial sector FFCO_2_ emissions were also only reported for natural gas consumption in Baltimore’s SRI, under the title ‘Commercial and Institutional Buildings and Facilities’. Emissions in Hestia-Baltimore were 276.0 (195.4–400.3) kt C for point and nonpoint commercial natural gas consumption while Baltimore’s SRI reported 130.3 kt C based on BGE utility data—entirely outside the 95% CI of Hestia-Baltimore with a difference of 71.7% for the central emission scenario. The extent to which large point sources are captured in the BGE data is not clear, and there may be differences between Hestia-Baltimore and the BGE utility data in the categorization of commercial and industrial emissions. These potential differences could not be diagnosed with the available data. The sum of commercial and industrial natural gas consumption emissions in the 2014 SRI was 285 kt C—only 6.4% less than the central Hestia-Baltimore emission estimate. Again, the lack of emissions associated with petroleum in Baltimore’s SRI represents a data gap, as petroleum fuel consumption was associated with 6.8% of commercial FFCO_2_ in Hestia-Baltimore in 2014.

#### Industrial

The industrial sector’s emissions of 175.3 (132.1–240.7) kt C in 2014 (11.8% of the city total) were dominated by point sources (79.1% of the industrial total), evident in Fig. 2 as relatively few grid cells with high emissions. The point sources occupied only sixteen grid cells out of 6066 grid cells that overlap with the city of Baltimore (0.2%). Nonpoint industrial buildings and processes accounted for the remaining 20.9%. The top 10% of emitting grid cells contained 89.9% of industrial emissions (Fig. 3). Unlike the commercial and residential sectors, petroleum was associated with most of the industrial FFCO_2_ (83.6%) while natural gas use made up the remaining 16.4%.

The industrial sector was difficult to compare between the SRI and Hestia-Baltimore due to apparent differences data sources and categorization. Again, only natural gas use was reported for ‘Manufacturing Industries and Construction’ emissions in Baltimore’s SRI. Baltimore’s SRI reported 155.6 kt C FFCO_2_ for natural gas use in this sector while the industrial natural gas use in Hestia-Baltimore was only 28.8 (22.1–38.8) kt C. Again, this discrepancy is likely due to differences in commercial and industrial building categorization between the SRI and Hestia-Baltimore. However, 83.6% of industrial emissions in Hestia-Baltimore were from petroleum fuel use in 2014—not natural gas. It is possible that some of the ‘Manufacturing Industries and Construction’ emissions in the 2014 SRI were categorized as (e.g.) commercial sector emissions in Baltimore-Hestia. Furthermore, the dominance of industrial point sources in Hestia-Baltimore highlights the need to include major point source facilities. Another category called Industrial Processes and Product Use (IPPU) was not reported in the SRI, which is allowed under the Global Protocol for Community-Scale Greenhouse Gas Emission Inventories (GPC) standard when data availability prevents an estimate.

### Electricity production

**Fig. 4:**
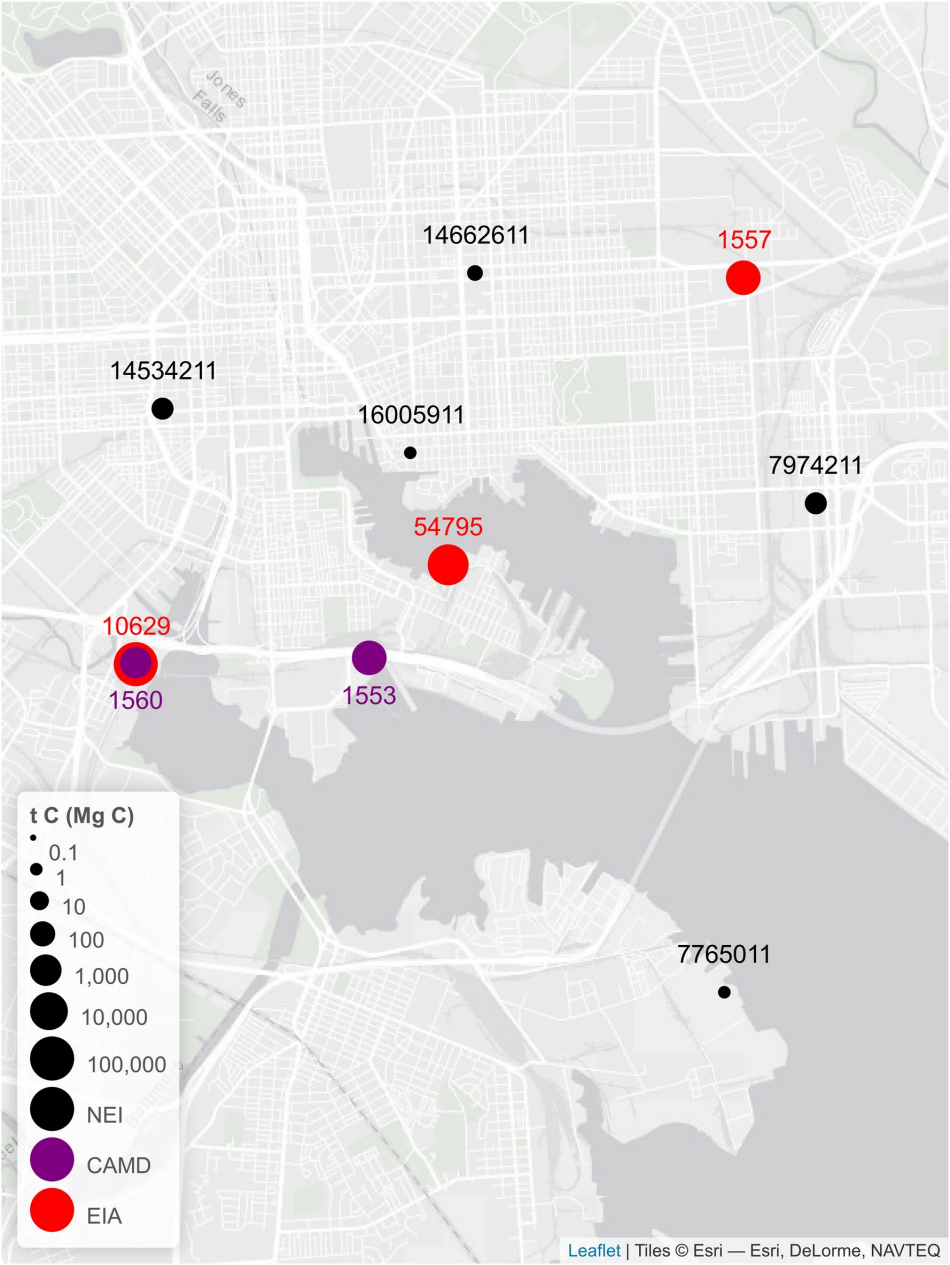
Electricity production emissions from NEI, CAMD, and EIA data for 2014. The units are metric tonnes of carbon (t C). The labels next to each facility indicate the ORISPL or EIS code (Table 3 and Table 4). Note that the four NEI facilities with no reported FFCO_2_ emissions are not shown. This map was made using R’s ‘leaflet’ package with the Esri World Gray Canvas basemap (Esri, DeLorme, NAVTEQ)

Scope 1 FFCO_2_ from electricity generation totaled 129.1 (112.3–145.9) kt C in 2014 (8.7% of the city total), making it the fifth largest sector from 2011 to 2015. Petroleum combustion was responsible for 75.5% of these emissions while the remaining 24.5% was derived from natural gas combustion. Specifically, Hestia-Baltimore contains ten facilities that reported non-zero FFCO_2_ emissions within the city of Baltimore. CO_2_ emissions from biofuel-based energy generation are not included in Hestia-Baltimore. No emissions from coal use are reported for this sector within Baltimore. Figure 4 shows the electricity producing point sources, which are clustered near the central core of the city and around the harbor. Baltimore contains two facilities that report hourly emissions to the Clean Air Markets Division (CAMD) which burn natural gas (3.7% of electricity production emissions in 2014). Three facilities that burn natural gas and/or petroleum have emissions estimated from monthly fuel consumption data from the Energy Information Administration in the US Department of Energy (DOE/EIA, 96.3% of sector total in 2014). Table 3 shows the central estimate of FFCO_2_ emissions from the CAMD and EIA facilities from 2010 to 2015. Additionally, nine electricity producing facilities are reported in the 2011 NEI point sector, though only five of these report non-zero emissions (less than 0.1% of sector total in 2014). Table 4 shows emissions for these facilities in 2014. The Wheelabrator Baltimore Refuse facility is the largest producer of FFCO_2_ from electricity production in the city (71% of Scope 1 power plant emissions and 6.2% of city total emissions in 2014), with FFCO_2_ emissions coming from petroleum-based solid waste, which is included in Baltimore-Hestia due to the fossil-fuel origin of the waste. However, the facility’s actual CO_2_ emissions are much higher due to additional combustion of biogenic solid waste [29].

**Table 3:** Electricity producing facilities from CAMD and EIA data within Hestia-Baltimore. Central emission estimates are reported in tonnes of carbon (t C)

ORISPL ID^a^	Facility name	Latitude	Longitude	Source	2010	2011	2012	2013	2014	2015
1553	Gould Street	39.2665	− 76.6042	CAMD	4491.2	4579.8	7187.6	3160.7	3243.6	3987.7
1560	Westport	39.2661	− 76.6297	CAMD	1873.1	101.5	2295.1	4931.8	1479.1	5324.5
1557	Philadelphia	39.2986	− 76.5636	EIA	1262.0	1808.3	501.7	1299.4	4797.7	1468.3
10,629	Wheelabrator Baltimore Refuse	39.266041	− 76.629653	EIA	76,609.5	88,883.6	87,925.1	89,984.0	91,614.4	92,094.4
54795	Domino Sugar Baltimore	39.2744	− 76.5956	EIA	31,498.4	26,862.1	26,800.7	26,862.3	27,841.2	28,627.3

^a^The Office of Regulatory Information Systems Plant Location (ORISPL) IDs were used to identify unique electricity producing facilities in the EIA and CAMD data

**Table 4:** Electricity producing facilities in Hestia-Baltimore from the 2011 NEI point reporting, scaled to 2014

Facility ID (EIS)^a^	Facility name	Latitude	Longitude	FFCO_2_ (t C)
14534211	University of Maryland-Baltimore	39.287562	− 76.626678	43.7
7974211	Complementary Coatings Corp.	39.279614	− 76.555723	42.6
14662611	Johns Hopkins Univ-Madison St	39.298954	− 76.592817	2.1
7765011	Sasol North America Inc	39.238333	− 76.565556	1.6
16005911	Trigen Energy-Inner Harbor East	39.283766	− 76.599694	0.8
7946811	GAF Materials Corporation	39.276062	− 76.554512	0
7946911	Grace-Davison Chemical	39.214167	− 76.570556	0
14534311	Baltimore Sun-East Cromwell Street	39.2625	− 76.577778	0
16004611	Verizon Communications	39.293022	− 76.615368	0

Central emissions are reported in metric tonnes of carbon (t C). The interannual scaling was performed using ratios of fuel usage from the EIA [16]

^a^The facility IDs used for NEI electricity production sources were associated with the USEPA’s Emissions Inventory System (EIS, https://www.epa.gov/air-emissions-inventories/emissions-inventory-system-eis-gateway)

Baltimore’s SRI contains emissions for several electricity producing facilities, though only six of these facilities were located within the city limits of Baltimore (Table 5). Electricity consumption for the other facilities should be quantified in Scope 2 emissions and therefore are not included in our comparison with Hestia-Baltimore. Of the six facilities in Baltimore’s SRI within the city boundary, five overlapped with Hestia-Baltimore facilities, though the Trigen Leadenhall St facility was categorized as an industrial point source in Hestia-Baltimore with FFCO_2_ emissions of zero in all 6 years. The remaining facility (Trigen North Central Ave) was not included in the Hestia-Baltimore data. The reason for the differences in emissions for the remaining facilities is not clear, though it is likely due to different data sources—CAMD CO-_2_ data and EIA fuel consumption in Hestia-Baltimore vs. USEPA Flight Database in the SRI. The difference is especially notable for the Wheelabrator facility, which is the largest electricity producing facility within the city of Baltimore. Furthermore, the second largest facility in Hestia-Baltimore—the Domino Sugar facility—was not included in the SRI under electricity producing facilities, though it may have been included in the industrial or commercial sectors. Overall, Hestia-Baltimore estimated Scope 1 FFCO_2_ emissions of 129.1 (112.3–145.9) kt C from electricity producing facilities within the city boundary, while the SRI reported 103.0 kt C—a difference of 22.5% compared to the central emission estimate. Meanwhile, the SRI reported total Scope 1 powerplant emissions of 2186.7 kt C from facilities both within and outside of the city boundary.

**Table 5:** A comparison of electricity FFCO_2_ emissions between Baltimore’s SRI and Hestia-Baltimore

Electricity producing facility	Baltimore SRI for 2014 (CO_2_ in t C)	Hestia-Baltimore for 2014 (t C)	Relative Mean Difference (%)
Gould Street	3609.8	3243.6	− 10.7
Philadelphia	5216.9	4797.7	− 8.4
Westport	1449.3	1479.1	2.0
Wheelabrator Baltimore Refuse	75,773.5	91,614.4	18.9
Trigen Leadenhall St^a^	11,723.5	0	− 200
Trigen North Central Ave^b^	5194.0	N/A	− 200
Total	102,967.0	101,134.8	− 1.8%

Electricity producing facilities in Baltimore’s self-reported inventory (SRI) emissions for the year 2014 were matched to emissions from Hestia-Baltimore v1.6 from 2014. Note that some facilities that were included in the Hestia-Baltimore emissions (Tables 3 and 4), such as the Domino Sugar facility, are not included in this table, but may be included in other sectors in the SRI (e.g., industrial)

^a^Reported as an industrial point facility in Hestia-Baltimore, not an electricity producing facility, named ‘Veolia Energy Baltimore Heating, LLP-Spring Gardens’. No carbon monoxide emissions were reported for this facility in the 2011 NEI so there are no FFCO_2_ emissions in any year

^b^No matching facility was included in the Hestia-Baltimore data

### Onroad

**Fig. 5:**
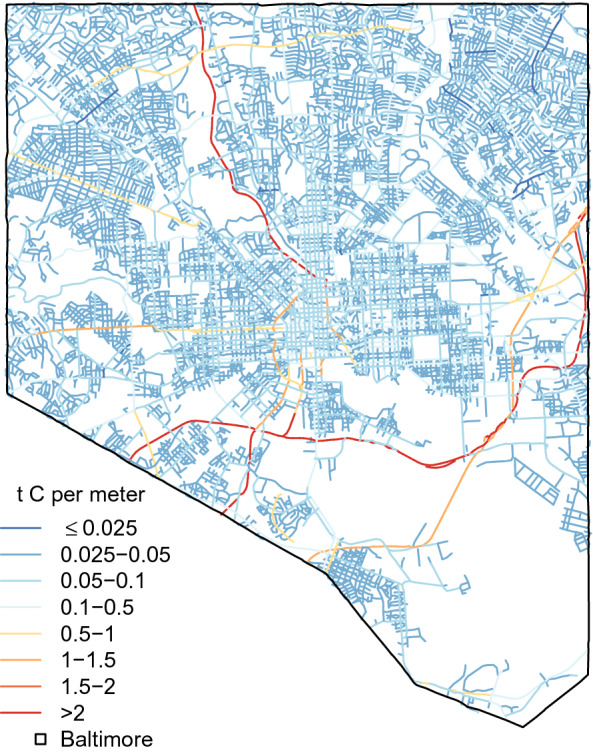
Annual onroad emissions on all road segments for 2014, normalized to unit length. Interstates and major arterial roadways appear in red and orange with more than 1 t C m^−1^ year^−1^, while roads in the downtown core of Baltimore generally had emissions of 0.1 to 1 t C m^−1^ year^−1^. Less densely populated parts of the county in the north and along the eastern and western edges had many roads with less than 0.1 t C m^−1^ year^−1^. This map was made in R

The onroad sector was the largest individual Hestia-Baltimore emission sector, with 508.9 (436.7–581.2) kt C in 2014 (34.2% of the city total). Gasoline usage accounted for 82.2% of onroad FFCO_2_ while diesel fuel represented the remaining 17.8%. Figure 5 shows the 2014 annual onroad emissions for all road segments in the city, normalized to segment length. Emissions along interstates and major arterial roadways were distinct among a backdrop of roadways with generally lower emissions. The downtown core of Baltimore also had higher emissions than the less densely populated suburban outskirts in the northern part of the city and along the eastern and western boundaries. The top 10% of emitting grid cells accounted for 43.2% of onroad emissions (Figs. 2, 3) while the 10% of road length with the highest emissions per meter were responsible for 54.0% of emissions (Fig. 5).

The difference between the Hestia-Baltimore onroad emissions and the city’s SRI estimate was small relative to other categories reported here, with Hestia emissions of 508.9 (436.7–581.2) kt C in 2014 and 589.6 kt C in the SRI—a difference of − 14.7% compared to the central emissions estimate and outside of Hestia-Baltimore’s 95% CI. The 2014 SRI provides estimates of ‘vehicle miles travelled’ (VMT) from the USEPA’s MOVES model, which are combined with vehicle-class-specific emission factors to estimate emissions. Hestia-Baltimore also uses data from the MOVES model, but instead of VMT, the FFCO_2_ output from the MOVES model in the 2011 NEI is used directly. Thus, the difference in the total onroad emissions are likely due to differing estimates of (e.g.) traffic volume and/or emission factors used in versions of the MOVES model. Nonetheless, Hestia-Baltimore’s distribution of onroad emissions to all road segments within the city provides insight into the high-emission onroad corridors that should be considered for sustainable transportation planning.

### Commercial marine vessels

For the years 2011 to 2015, commercial marine vessels (CMV) emissions within ports were the sixth largest FFCO_2_ emission sector in Baltimore, behind Scope 1 electricity generating sources. Note that no ‘underway’ shipping (shipping lanes along coast or towards open water) emissions were included within the city boundary as these emissions were allocated to Baltimore County in the 2011 NEI. FFCO_2_ emissions totaled 63.4 (42.8–95.8) kt C in 2014, representing 4.3% of the city total. However, CMV emissions in 2010 were 431.3 (291.2–652.1) kt C, or 24.0% of the city total, only behind the onroad sector with 511.7 (439.0–584.3) kt C (28.5% of the city total). The maximum in 2010 reflects annual fuel sales data from the EIA ‘vessel bunkering use’ and residual fuel oil sales for transportation [16]. CMV emissions were not reported in the SRI.

### Aircraft, nonroad, and rail

The nonroad, rail, and aircraft sectors cumulatively represented slightly more than 2% of the city’s total emissions in 2014. Nonroad emissions were the largest sector among the three with 27.2 (26.1–28.2) kt C in 2014 (1.8% of the city total), while rail emissions (4.0 (3.3–5.2) kt C, 0.3% of the city total) and aircraft emissions (0.6 (0.5–0.9) kt C, < 0.1% of the city total) were the smallest emitting sectors within the city. The relatively low aircraft emissions reflect the fact that the Baltimore/Washington International Airport (BWI) lies within neighboring Anne Arundel County and is therefore not included in the Scope 1 Hestia-Baltimore emissions.

Scope 1 emissions from these sectors were not included the SRI—a practice which is compliant with the GPC protocol when data are not available. Several of these sources are outside of the regulatory purview of the city, but are Scope 1 FFCO_2_ emissions nonetheless. Emissions from aircraft and rail both accounted for less than 1% of emissions in Hestia-Baltimore. However, nonroad emissions account for nearly 2% of FFCO_2_ in Hestia-Baltimore. Again, the USEPA NEI serves as an indirect source of FFCO_2_ emissions in the absence of centralized data (e.g. from a utility).

## Discussion

### Hestia-Baltimore results

The total FFCO_2_ emissions in Baltimore were dominated by onroad vehicles and stationary building/point source emissions (residential, commercial, and industrial sectors) in all years except for 2010, when the CMV sector was the second largest emitting sector after onroad. This departure from other years was the result of state-scale fuel sales data, used to scale emissions from the base year of 2011 (“Method**s**” section), exhibiting a large decline from 2010 to 2011 and beyond. Gasoline emissions dominated the onroad sector, where only 10% of the road surface length contributed to more than half of the onroad emissions. This result underscores the potential emissions reductions associated with traffic patterns on major arterial roadways (Fig. 5).

The second and third largest sectors in all years except for 2010 were commercial and residential, respectively. Both of these sectors were dominated by natural gas consumption, though petroleum fuel consumption was associated with non-negligible FFCO-_2_ emissions in both sectors (6.8% of commercial and 13.4% of residential emissions in 2014). Residential emissions were assigned entirely to nonpoint buildings while nonpoint commercial building emissions accounted for 77.3% of the sector total in 2014. This result highlights the importance of building energy efficiency in Baltimore’s FFCO_2_, as the nonpoint commercial and residential emissions represented 34.4% of the city’s total emissions in 2014 and much of the city’s building stock is several decades old (see Additional file 1). Building stock has been identified as a key infrastructural component in urban GHG mitigation [30, 31].

The industrial sector deviated from the commercial and residential sectors as 83.6% of FFCO_2_ emissions in 2014 were from petroleum fuel consumption. Industrial point sources were responsible for 79.1% of total industrial emissions in 2014. This result was markedly different than the nonpoint-dominated commercial sector. Industrial point sources alone represented 9.3% of total FFCO-_2_ emissions in Baltimore in 2014.

Scope 1 power plant emissions summed to less than 10% of city total emissions in all years, with the Wheelabrator facility dominating emissions in this sector, followed by the Domino Sugar facility. The Wheelabrator facility may be shut down in the near future due to concerns of air quality [32], which would reduce the city’s Scope 1 FFCO_2_ emissions. However, the loss of electricity generation within the city would increase the demand for transboundary power imports from neighboring counties, thereby increasing the city’s Scope 2 emissions. Even if the facility is shut due to air quality concerns, sustainability efforts related to electricity consumption should focus on Scope 2 consumption to reduce that Baltimore’s system-wide GHG footprint [33].

Baltimore-Hestia contains Scope 1 FFCO_2_ emissions from several additional sectors. Emissions from the CMV sector were entirely categorized into the ‘port’ subsector, with no emissions reported for underway shipping. This sector was highly variable, accounting for 4.3% (2014) to 24.0% (2010) of the city’s total emissions. Nonetheless, CMV emissions represent a non-negligible source of FFCO_2_ within Baltimore. Nonroad emissions accounted for less than 2% of the total emissions in each year, while rail emissions were less than 0.3% and aircraft emissions were less than 0.2%. While nonroad, rail, and aircraft emissions are not large relative to the city total, they may intersect with other sustainability priorities within the city [34, 35].

Scope 1 FFCO_2_ emissions from Hestia-Baltimore totaled 1487.3 (1158.9–1944.9) kt C in 2014. When sources that are not included in the SRI are excluded from Hestia-Baltimore, the totals are very similar—1159.4 kt C for Hestia-Baltimore and 1182.6 kt C for the SRI—a difference of − 2.0% compared to the central estimate (Tables 5 and 6). However, these estimates are more than 300 kt C (20%) short of the central Hestia-Baltimore total Scope 1 FFCO_2_ emission for all sectors.

**Table 6:** Sectoral comparison of FFCO_2_ emissions in Baltimore’s SRI for 2014 and matching emissions from Hestia-Baltimore

Source	Baltimore SRI for 2014 (CO_2_ in t C)	Hestia-Baltimore for 2014 (t C)	Relative Mean Difference (%)
Residential natural gas	204,152	244,624	18.0
Commercial natural gas	130,299	275,977	71.7
Industrial natural gas	155,557	28,789	− 137.5
Onroad	589,595	50,8917	− 14.7
Total	1,079,603	1,058,307	− 2.0

Scope 1 electricity emissions for overlapping facilities are compared in Table 5

### Implications for Baltimore’s sustainability planning

Estimating urban GHG emissions is challenging for several reasons:

1. Inventory development is labor intensive and therefore expensive, and cities that operate with limited financial resources will struggle to build and maintain high-quality GHG inventories.
2. Data for FFCO_2_ sources are idiosyncratic among cities; varying urban typologies, data availability, and state/local policy can prevent cities from identifying and quantifying all sources of FFCO_2_ within their boundaries and beyond.
3. Users of GHG emissions estimates often have different motives. City governments often have limited policy levers and therefore may have a narrow range of emissions sources for which they have regulatory interest [36]. However, carbon cycle science requires all Scope 1 sources to be quantified.

Emissions estimates developed with varying assumptions, methods, and data sources lead to large differences in total Scope 1 emissions estimates and even larger differences within individual sectors. For nonpoint buildings and point sources, some fossil fuels may not be delivered by a central utility, and therefore relying only on utility data would lead to an underestimate of Scope 1 GHG emissions from nonpoint buildings. In Hestia-Baltimore, this problem is especially apparent in the industrial sector, where petroleum use was responsible for 83.6% of emissions in 2014. Baltimore’s SRI did not report petroleum use for the residential, commercial, or industrial sectors, likely due to a lack of data on private-sector transactions. However, state-level air quality reporting provides indirect estimates of energy usage for these sectors and fuels, often through proxies [16]. Cities may benefit by developing relationships with state-level air quality agencies to exchange energy usage data that is relevant to both air quality and GHG mitigation. Furthermore, IPPU emissions were not reported in the SRI, which is allowed under the GPC standard and reflects the city’s lack of policy levers to mitigate emissions from private industry. However, this exclusion contributes to an underestimate of industrial emissions as point sources accounted for 79.1% of industrial sector emissions in 2014 in Hestia-Baltimore. Likewise, large differences in individual power plant emissions were apparently driven by different input data sources between the SRI and Hestia-Baltimore, highlighting the need to identify best practices for estimating Scope 1 emissions from electricity production.

The difference in assumptions and data sources between Baltimore’s SRI and Hestia-Baltimore also demonstrates conflicting goals of GHG emissions estimates. First, many cities, including Baltimore, develop GHG emissions reduction goals as part of a broader sustainability effort within the city, ultimately culminating in city-led actions to reduce emissions and lessen other environmental and social impacts [34, 35]. Within that context, excluding sectors for which the municipal government has no policy levers—e.g., established point facilities that are compliant with the Clean Air Act, or airports where most travelers do not reside in the city—allows the city to focus on the emitting sources that can by influenced by city-level policy. Furthermore, SRI development allows for the inclusion of Scope 2 and Scope 3 GHG footprinting and mitigation planning through (e.g.) decarbonizing the electricity mix brought into the city from power plants in other counties or states. Such scopes allow for differing urban typologies to account for trans-boundary GHG emission footprinting [33].

However, city-level inventories often contain substantial uncertainties and can therefore benefit from independent evaluation for each Scope and gas. Without this evaluation, errors and exclusions in GHG emissions estimates may result in financial resources being directed towards mitigating GHG sources that are relatively insignificant in the broader context of a city’s GHG footprint while other important sources remain unchecked. Furthermore, ongoing inventory evaluation can ensure that policies are having the intended impact on atmospheric GHG levels and provide a course-correcting feedback to policymakers [37]. For Scope 1 emissions, the use of atmospheric monitoring within a city combined with models of atmospheric transport and space/time-resolved data products, such as Hestia, are considered the state-of-the-art in terms of independent evaluation. Hence, the generation of a Scope 1 SRI estimate, avoiding conflation with Scope 2 or Scope 3 emissions is crucial to avail of this evaluation capacity, though we emphasize that Scope 2 and 3 emissions are also critical needs for urban policymakers to reduce system-wide GHG emissions [33]. By excluding large Scope 1 emissions sources—e.g. industrial petroleum use and CMV emissions in Baltimore—emissions estimates cannot be evaluated through atmospheric monitoring, leaving city planners with no choice other than to plan, prioritize, and fund city-led mitigation efforts using potentially erroneous and incomplete Scope 1 GHG emissions estimates. Thus, while certain Scope 1 emission sectors/sources may not be of interest to city planners due to a lack of policy levers, they are still necessary for developing a comprehensive and evaluable estimate of a city’s total Scope 1 GHG footprint.

## Conclusions

The Hestia-Baltimore v1.6 FFCO_2_ emissions data product stems from Vulcan v3.0—a national-scale FFCO_2_ emissions data product with emissions disaggregated by sectors, fuel types, and processes [16]. The sectoral emission totals in the City of Baltimore were incorporated into the Hestia-Baltimore system and distributed in space and time using local data sources where available. The resulting Hestia-Baltimore v1.6 product is available on a 200 m resolution grid as total annual and hourly emissions for the years 2010 to 2015 [27]. Onroad, commercial, residential, and industrial emissions represent the largest emitting sectors in all years except for 2010, when CMV emissions were the second largest sector after nonroad. Aircraft and rail emissions each represented less than 1% of the totals, while electricity production, CMV (except for 2010), and nonroad sources each accounted for less than 10% of emissions.

A comparison of Hestia-Baltimore with the Scope 1 FFCO_2_ emissions in the city’s SRI for the year 2014 revealed several differences in data sources, methodology, and included/excluded Scope 1 emission sources. The SRI included Scope 2 and 3 emissions and Scope 1 emissions for other gases that were not included in Hestia-Baltimore. While petroleum fuel consumption accounted for 27.1% of residential, commercial, and industrial Scope 1 emissions in Hestia-Baltimore in 2014, petroleum was not included in the SRI, likely due to data limitations. For the residential sector, the central emissions estimate for natural gas consumption was 18.0% higher in Hestia-Baltimore than the SRI, though the SRI emissions fell within the 95% CI of Hestia-Baltimore. Likewise, only natural gas consumption was included in the SRI’s commercial and industrial sector, leading to relative mean differences of 71.7% for commercial and − 137.5% for industrial between the SRI and the central Hestia-Baltimore estimate. These differences may be due to differences in the categorization of commercial and industrial sources. Additionally, the exclusion of IPPU sources in the SRI, which is permitted by the GPC standard, represents a significant data gap.

Onroad emissions were the closest match between Hestia-Baltimore and the SRI as both estimates utilized the USEPA MOVES model. The observed difference may be due to different versions of MOVES output in Hestia-Baltimore and the SRI, though the specific reason could not be determined. Likewise, differences were found between the SRI and Hestia-Baltimore Scope 1 emissions in the electricity production sector due to different data sources. Emissions from the nonroad sector and Scope 1 rail and aircraft emissions were not included in the SRI, though Scope 2 electricity consumption in buildings and along electrified railways, as well as Scope 3 waste emissions, were included. CMV emissions, which were entirely associated with ports, were the sixth largest source in Hestia-Baltimore in 2011 to 2015 and the second largest source in 2010. However, no CMV or port emissions were included in the SRI. Like IPPU sources and other transportation sectors, CMV emissions are not required to be reported under the GPC standard. Overall, the sums of matching Scope 1 emission sources in Hestia-Baltimore and the 2014 SRI matched within 2% for the central estimate, though some individual sectors showed poor agreement. Furthermore, the sum of Scope 1 SRI emissions was more than 20% lower than all central Scope 1 emissions in Hestia-Baltimore.

These differences present challenges to city planners who are developing action plans to reduce GHG emissions. The data sources, assumptions, and categorical inclusions/exclusions used to develop emissions estimates may influence the development of climate action plans and the prioritization of GHG mitigation actions. Not all Scope 1 emission sources may be counted in a city’s SRI for its climate action plan due to policy lever constraints. However, all Scope 1 emissions within a city’s boundary should be quantified and distributed in time and space for the sake of evaluation using atmospheric monitoring. Without evaluation, erroneous data in Scope 1 GHG emissions estimates may lead to costly efforts to reduce emissions from sources that are actually small, while larger sources remain unidentified and therefore unmitigated. Furthermore, the combination of atmospheric monitoring and complete Scope 1 emissions in time and space allows for emissions mitigation efforts to be monitored for success. We emphasize that comprehensive, spatiotemporalized, and verifiable Scope 1 emission estimates that are not conflated with Scope 2 or Scope 3 emissions are a critical tool required for city planners to make informed and effective decision making in their GHG mitigation efforts.

## Methods

### Study domain

The City of Baltimore (also referred to as Baltimore City or simply Baltimore) is located on the western shore of Chesapeake Bay in the state of Maryland. Baltimore County borders the city on the east, north, west, and southwest sides, though Baltimore City is independent from Baltimore County—thus, the emissions in Hestia-Baltimore represent emissions for Baltimore City and not Baltimore County. The mouth of the Patapsco River—a tidal estuary inlet—lies in the southern half of the city, immediately south of the city’s downtown area, with numerous inlets along the southern part of the city. The southernmost part of the city lies along the southern side of the Patapsco River. The city’s population was 620,961 in the 2010 US Census and has declined from a peak population of ~ 950,000 in the year 1950 [38].

### Input data

The initial data sources for Hestia-Baltimore v1.6 emissions are sectoral, county-scale FFCO_2_ output from Vulcan v3.0. Full details on data and methodology for Vulcan, including references and links to datasets, are available in Gurney et al. [16] and are not described in full detail here. Instead, a brief overview of data and methods is provided for Vulcan output. City- and state- specific data for relevant sectors in the Hestia-Baltimore product are described below, with additional details in the Additional file 1. Generally, Vulcan provides a space/time-explicit FFCO_2_ emissions estimate across the US landscape based on local data sources (points, lines and polygons) that have been archived at the federal level—notably within the USEPA NEI. Hestia is considered to be nested within Vulcan, and emissions from Vulcan are further downscaled in space and time in addition to location correction (for point sources such as powerplants and large industrial facilities) where additional local data is available. Spatial downscaling is performed for buildings in the residential, commercial and industrial sectors from the county totals in Vulcan to the building/parcel spatial scale. The onroad emissions are downscaled in time from the annual to hourly scale with local traffic data. All other emission sources (rail, nonroad, airports, commercial marine vessels) are passed directly from Vulcan to the Hestia landscape. Hence, Both Vulcan and Hestia are bottom-up, process-based emissions estimates. But Hestia further downscales the space/time distribution and corrects locations of point sources, where necessary. In some Hestia domains (e.g. the Los Angeles Megacity) additional local data allows for a re-estimation of emissions in, for example, specific point sources. In this sense, the exact Hestia methodology varies somewhat from city to city. For Hestia-Baltimore, the local data used to redistribute Vulcan emissions are individual building footprints and data local traffic data, and corrected geolocations of point sources.

The conservatively estimated upper and lower 95% confidence interval bounds for the emissions in Hestia are identical to the bounds in Vulcan, which are also discussed in Gurney et al. [16]. The variables that contributed to uncertainty were the reported CO and CO_2_ emissions in the 2011 NEI, emission factors, and estimates of fuel and energy consumptions. Currently, this uncertainty is represented at all space/time scales through the aggregation of the sector/fuel uncertainties applied to the individual emitting processes. Hence, the uncertainty will vary in space depending upon the mixture of fuel/sector representation in any given location. The spatial and temporal uncertainties have not yet been incorporated into the Vulcan methodology, but will be in future versions. Additional errors are likely introduced into Hestia as a result of the redistribution from Vulcan to the higher-resolution representation in Hestia. These errors have not been included in the current uncertainty treatment but will be incorporated in future work.

Vulcan utilizes CO emissions from the 2011 NEI for several sectors—nonpoint (commercial, industrial, and residential), point (mostly commercial and industrial), airport/helipad, rail, nonroad, commercial marine vessels (CMV), and some electricity production. Most of the electricity production FFCO_2_ data come from the Clean Air Markets Division (CAMD), with additional emissions estimated from Department of Energy/Energy Information Administration (DOE/EIA) fuel consumption. The onroad emissions come directly from the MOVES model results in the 2011 NEI. Each of these sectors is distributed in space and time using surrogate spatiotemporal information, where available. Notably, nonpoint building-sector emissions are distributed at the scale of census block groups and traffic data are distributed to a national road basemap using federal-level traffic data. While Vulcan includes CO_2_ emissions from clinker production at cement plants, no emissions associated with cement fall within the Baltimore City boundary.

In Hestia-Baltimore, emissions from nonpoint buildings in the commercial, industrial, and residential sectors were spatially distributed to parcel and building footprints obtained from the Maryland Department of Planning [39] and the Baltimore City Government [40]. The non-electric energy use intensity (NE-EUI) was calculated for building types and vintages and multiplied by each building’s floor area to estimate the buildings’ relative FFCO-_2_ footprint within the city. Hourly emission profiles follow the Vulcan v3.0 methodology. Additional details on the spatial distribution are available in Additional file 1: Text S1. Likewise, onroad emissions within the city are distributed in space and time using local traffic data. The spatial distributions of average annual daily traffic (AADT) follow Vulcan v3.0 methodology, while the hourly profiles are distributed using kriging for midweek days (Tuesday through Thursday) and are evenly distributed during the remaining weekdays. The temporal allocation is reviewed in more detail in Additional file 1: Text S2 and shown in Additional file 1: Figure S1. All other sectors are distributed in space and time following the Vulcan v3.0 procedures except for airports/helipads, which are assigned a flat temporal distribution in Hestia-Baltimore.

### Comparing Hestia-Baltimore with self-reported emissions

The most recent and complete emissions data for the city of Baltimore are available from the 2014 SRI (2020 email from L. McNeilly to S. M. Miller and G. S. Roest, unreferenced, see notes). This inventory was made using the Global Protocol for Community-Scale Greenhouse Gas Emission Inventories (GCP) City Inventory Reporting and Information System (CIRIS) spreadsheet tool, which allows cities to quantify Scope 1, 2, and 3 emissions of CO_2_, methane, and nitrous oxide. The emissions included in Hestia-Baltimore and the city’s SRI are compared in Table 2, while the Scope 1 CO_2_ emissions are compared in detail in Tables 5 and 6. Emissions in the SRI were disaggregated into individual gases, so the CO_2_ emissions in Tables 5 and 6 do not include other GHGs. The CO_2_ emissions in the SRI, reported as metric tonnes of CO_2_, were converted to metric tonnes of carbon (t C).

We emphasize that neither the 2014 SRI nor Hestia-Baltimore are considered to be “correct” or “incorrect”—instead, the observed differences represent varying data sources, methodologies, assumptions, and decisions to categorically include or exclude emissions based on the purpose of the emissions estimate. A review of each sector is provided in  Results” section, followed by a discussion of important takeaways from this comparison. The differences reported in  “Results” section and in Tables 5 and 6 represent the relative mean difference (RMD):

1 $$ RMD = 100\% \times \frac{{H_{s} - SRI_{s} }}{{\frac{1}{2}\left( {H_{s} + SRI_{s} } \right)}} $$

where *H*_*s*_ represents the Hestia-Baltimore emissions for the subset of sources based on the sector, fuel type, or other identifier, and *SRI*_*s*_ represents the self-reported emissions for the same subset of sources. A positive RMD indicated that Hestia-Baltimore Scope 1 emissions for a particular sector and fuel are higher than the equivalent emissions in the SRI.

Point and nonpoint petroleum/coal emissions, rail, aircraft, nonroad, and electricity production emissions are not included in the comparison in Table 6 due to inconsistent overlap between the SRI and Hestia-Baltimore, preventing comparisons between these sectors. Baltimore’s SRI emissions for the residential sector were based on metered natural gas consumption at single- and multi-family homes. The Baltimore SRI data for the commercial sector included ‘Small Commercial Natural Gas’, ‘General Service Large Natural Gas’, and ‘General Serv—Daily Natural Gas’ for ‘Commercial and institutional buildings and facilities’. In the industrial sector, the Baltimore SRI emissions included ‘Interruptible Service Natural Gas’, ‘Patapsco and Back River Natural Gas Consumption’, and ‘Montebello and Ashburton Natural Gas Consumption’ for ‘Manufacturing industries and construction’. Lastly, onroad emissions in Baltimore’s SRI were disaggregated by vehicle class, not fuel type. Both Baltimore’s SRI and Hestia-Baltimore onroad emissions are based on the sum of vehicle class emissions and fuel type emissions, respectively.

The electricity producing facilities in Table 5 are the facilities reported in Baltimore’s 2014 SRI that fall within the city limits of Baltimore. Electricity consumption from facilities outside of the city’s limits should only be considered Scope 2 [7, 8] and therefore are not included in Hestia-Baltimore Scope 1 FFCO_2_ emissions. Only FFCO_2_ emissions are included in the comparison in Table 5. Emissions of biogenic CO_2_ from organic waste were reported in Baltimore’s SRI but were not include in Table 5. Electricity producing facility emissions in Baltimore’s 2014 SRI were collected from the USEPA Flight Database.

## Supplementary information

**Additional file 1.** Detailed methodology and information for the distribution of nonpoint building emissions and onroad emissions.

## Data Availability

The data that support the findings of this study are openly available in the National Institute of Standards and Technology (NIST) Public data repository at 10.18434/T4/1503342. Baltimore’s 2014 self-reported greenhouse gas inventory was made available through a personal email communication between Lisa McNeilly at the Baltimore Office of Sustainability (https://www.baltimoresustainability.org/, sustainability@baltimorecity.gov).

## Abbreviations

AADT: Average annual daily traffic
ACES: Anthropogenic Carbon Emissions System
BGE: Baltimore Gas and Electric
CAMD: Clean Air Markets Division
CI: Confidence interval
CIRIS: City Inventory Reporting and Information System
CMV: Commercial marine vessel
CO: Carbon monoxide
CO_2_: Carbon dioxide
CO_2_e: Greenhouse gas emissions measured as carbon dioxide equivalents
DARTE: Database of Road Transportation Emissions
DOE: US Department of Energy
EIA: Energy Information Administration
FFCO_2_: Fossil-fuel carbon dioxide
GCMCE: Global Covenant of Mayors for Climate & Energy
GHG: Greenhouse gas
GPC: The Global Protocol for Community-Scale Greenhouse Gas Emission Inventories
ICLEI: Local Governments for Sustainability
INFLUX: Indianapolis Flux Experiment
IPPU: Industrial Processes and Product Use
MOVES: MOtor Vehicle Emission Simulator
NE-EUI: Non-electric energy use intensity
NEI: National Emissions Inventory
ORISPL: Office of Regulatory Information Systems Plant Location
RMD: Relative mean difference
SRI: Self-reported inventory
USEPA: US Environmental Protection Agency
VMT: Vehicle miles travelled
