# Supplementary material for: Informing urban climate planning with high resolution data: the Hestia fossil fuel CO2 emissions for Baltimore, Maryland
Source: Carbon Balance Manag. 2020 Oct 14;15:22. doi: 10.1186/s13021-020-00157-0 (PMC7559750; doi:10.1186/s13021-020-00157-0)
Supplement: Supplementary file 1 — Additional file 1. Detailed methodology and information for the distribution of nonpoint building emissions and onroad emissions. [file 13021_2020_157_MOESM1_ESM.pdf]

Supporting information for:

Informing urban climate planning with high resolution data: the Hestia fossil fuel CO_2 emissions for Baltimore, Maryland

Geoffrey S. Roest[a]*, K. R. Gurney[a], S. M. Millier[b], and J. Liang[c]

[a] *School of Informatics, Computing, and Cyber Systems, Northern Arizona University, Flagstaff, AZ, USA;*

[b] *Department of Environmental Health and Engineering, Johns Hopkins University, Baltimore, MD, USA;*

[c] *School of Life Sciences, Arizona State University, Tempe, AZ, USA (Now at ESRI)*

*Corresponding author; geoffrey.roest@nau.edu

Contents

Text S1: Detailed methods for the spatial distribution of nonpoint building emissions to building and parcel footprints

Text S2: Detailed methods for the temporal distribution of onroad emissions to individual road segments

Figure S1: Annual average temporal distribution for particular days of the week as derived from Baltimore Metropolitan Council traffic data.

Table S1: A crosswalk between the Maryland and Baltimore governments' parcel and building data descriptions and the CBECS/RECS/MECS building descriptions.

Table S2: Crosswalk between CBECS building types and DOE2/eQUEST building types.

Table S3: Crosswalk between RECS building types and DOE2/eQUEST building types.

References

Text S1. Nonpoint buildings

$FFCO_2$ emissions for residential, commercial, and industrial nonpoint buildings represent on-site combustion processes only and not associated with large emissions allocated to a stack (which are categorized as point emissions). Emissions associated with the consumption of electricity in buildings are spatially allocated to electricity generating units (i.e. power plants) and are not included in this sector. Annual emissions for the city followed Vulcan v3.0 methodology (1). The hourly temporal profiles followed the same methods as well, but hourly emissions were assigned to individual parcels and buildings instead of US Census block groups. Local data are used to distribute the city-level $FFCO_2$ emissions from Vulcan v3.0 in space. Parcel and building data for the city of Baltimore were obtained from the Maryland Department of Planning (2). Attributes for parcels and buildings include the land-use sector (residential, commercial, industrial, and exempt), building style, building description, structure floor area, and year built. Some records include non-building descriptions (e.g. "AUTO parking lot"). Additional building data from the Baltimore City Government (3) were used to gap fill missing data - notably in the structure floor area and year built columns - though this dataset only extended to the year 2007, while the state-wide dataset extended to 2015. This city-specific dataset contained complete records for structure floor area, but was otherwise less descriptive. The combination of the state and city data were used for structure floor areas.

As building design and codes have changed over time, buildings have generally become more energy efficient in recent years (4). Therefore, building stock age plays a role in the spatial distribution of nonpoint emissions. The "year built" data in the parcel and building datasets from the state of Maryland and the city of Baltimore represent the date that the primary structure was built. For missing data, the sector-specific mean was

used to estimate the year built (residential: 1949, commercial: 1951, industrial: 1968). No data were available regarding retrofitting of older buildings to more recent building codes - hence, the year a building was built was used to define its energy use intensity.

Energy use in buildings was based on data from the EIA's Commercial Building Energy Consumption Survey (CBECS), Residential Energy Consumption Survey (RECS), and Manufacturing Energy Consumption Survey (MECS). The "Microdata" building survey data contain building characteristics for multiple survey years, with data closest to the core year of 2011 being used in Hestia. These survey data contain information used to calculate the non-electric energy usage intensity (NE-EUI) for building prototypes, specific to fuel type (natural gas and liquid "petroleum" fuels) and US Census Regions. Baltimore falls under the South Atlantic (Division 5). Note that the calculated NE-EUIs used in Hestia represent averages in the Census Region for fuel types, and that the actual NE-EUI will vary for individual buildings due to (e.g.) building design, decay, retrofitting, microclimate, and use by occupants. No data exist on the natural gas pipeline system in Baltimore at the building scale, nor the distribution of liquid fuels to individual buildings. Therefore, natural gas and petroleum usage is assumed to be distributed to all nonpoint buildings in Baltimore based on their NE-EUI.

Unlike other Hestia cities – Indianapolis (5), Salt Lake City (6), and Los Angeles (7) – the building classification scheme of CBECS and RECS were used instead of a Hestia-specific building scheme. The CBECS and RECS building classifications are also matched to the building types in the eQUEST model, which was used for temporal emissions allocation. Table S1 shows a crosswalk between CBECS/RECS building types and state/city parcel and building data. Tables S2 and S3 show crosswalks between the eQUEST building types and the CBECS and RECS building types, respectively. Furthermore, the age of buildings in Baltimore were

categorized into building vintages within the CBECS, RECS, and MECS data. However, due to the sample sizes, building vintages in Hestia were represented by broad age ranges. For commercial and residential buildings, NE-EUIs were calculated for two vintages - pre-1980 and post-1970. No vintages were assigned to industrial buildings due to a lack of data.

The 2012 CBECS microdata were used for the commercial sector. The data contain the building floor area and energy consumption (natural gas and fuel oil/diesel/kerosene - collectively referred to as “petroleum” in Hestia), along with a weighting factor that converts individual building sampling into a population total. For each Census division, building type, and vintage, the NE-EUI for natural gas ($NEEUI_{NG}$) is achieved by summing the weighted natural gas use for each building and dividing by the sum of the weighted floor areas for each building:

$$NEEUI_{NG,i,j,k} = \frac{\sum_m w_m \times N_m}{\sum_m w_m \times A_m} \quad \text{Eq. 1}$$

where *i* is the Census division, *j* is the building type, *k* is the vintage, *m* is the record in the dataset (within Census division *i*, building type *j*, and vintage *k*), N_m is the natural gas consumption for each building (kBTU), A_m is the floor area of each building (ft^2), and w_m is the weighting factor that converts individual sampling to population totals. The same procedure is followed for fuel oil/diesel/kerosene consumption ($NEEUI_{FK}$). A similar procedure is followed for residential buildings. In the RECS data, the 2009 survey data were used. An equation identical to Eq. 1 is used for the same census division and the same fuel categorization (natural gas and “petroleum”) and the same vintages.

The MECS data for 2010 were used for industrial buildings. The MECS data

represent the sum of sampled buildings specific to Census division and manufacturing sector, represented by North American Industry Classification System (NAICS) codes, as opposed to individually sampled buildings within building types for CBECS and RECS. Thus, the fuel-specific (natural gas and the sum of residual fuel oil and distillate fuel oil) were calculated as follows:

$$NEEUI_{NG,i,j} = \frac{N_{i,j}}{A_{i,j}} \quad \text{Eq. 2}$$

where *i* is the Census division, *j* is the manufacturing sector, *N* is the natural gas consumption, and *A* is the floor space. The NE-EUI for "petroleum" fuels is calculated in the same manor.

Each parcel or building is assigned an annual $FFCO_2$ emission, *E(b)*, based on the NE-EUI, the structure floor area, and the county-wide emissions:

$$E(b)_s^f = E_{tot} \times \frac{NEEUI_f^s \times A(b)}{\sum_b NEEUI_f^s \times A(b)} \quad \text{Eq. 3}$$

where *b* represents each individual structure, *f* represents the fuel type (natural gas or "petroleum"), *s* represents the building sector, *NEEUI* represents the fuel- and building-type-specific NE-EUI, *A* represents the structure floor area, and E_{tot} represents the total county $FFCO_2$ emissions. For coal, however, the NE-EUI is assumed to be 1 for all buildings since no building survey data on coal consumption exist. Thus, $FFCO_2$ from coal consumption is proportional to floor area.

Text S2. Onroad emissions

Onroad emissions began with output from Vulcan v3.0 (1). Briefly, county-level $FFCO_2$ emissions were obtained from the USEPA's MOVES model which is included in the 2011 NEI. Emissions are reported by 13 vehicle types and 12 road types, which are further aggregated in Vulcan to match road map and traffic count data. The spatial distribution of $FFCO_2$ to road segments followed the Vulcan v3.0 methodology, while the hourly distribution used local traffic count data from the Baltimore Metropolitan Council (8). In 2014, there were 113 traffic counting stations within the city of Baltimore. Due to incomplete data, annually averaged diurnal profiles were created for days of the week. This method captures the diurnal and weekly traffic cycles, though seasonal changes in traffic patterns are not characterized. For midweek (Tuesday, Wednesday, and Thursday) traffic patterns, enough data existed to justify a spatial interpolation through kriging. Traffic counts for each hour of the day were averaged at each station across the mid-week days (Tuesday, Wednesday, and Thursday) and distributed to 24 hourly shapefiles, each with 113 points for each station. These data were kriged within the city boundary to create a raster surface of interpolated traffic counts for each hour. Road segments within the city were assigned hourly traffic counts by intersecting the road segments with the raster cells, thereby creating a 24-hour profile for each road segment in the city for midweek days. Fridays through Mondays each have unique traffic patterns associated with typical non-work-week traffic patterns. These days did not have enough data to justify kriging. Instead, average diurnal profiles for each of these weekdays were developed with the available data and applied to all road segments in the city. While this method does not capture spatial variability in traffic patterns or seasonal variations, the profiles are similar to the state-level time profiles used in Vulcan, lending confidence to the method. Supporting figure S1 shows

the hourly profiles for Friday through Monday.

Figures

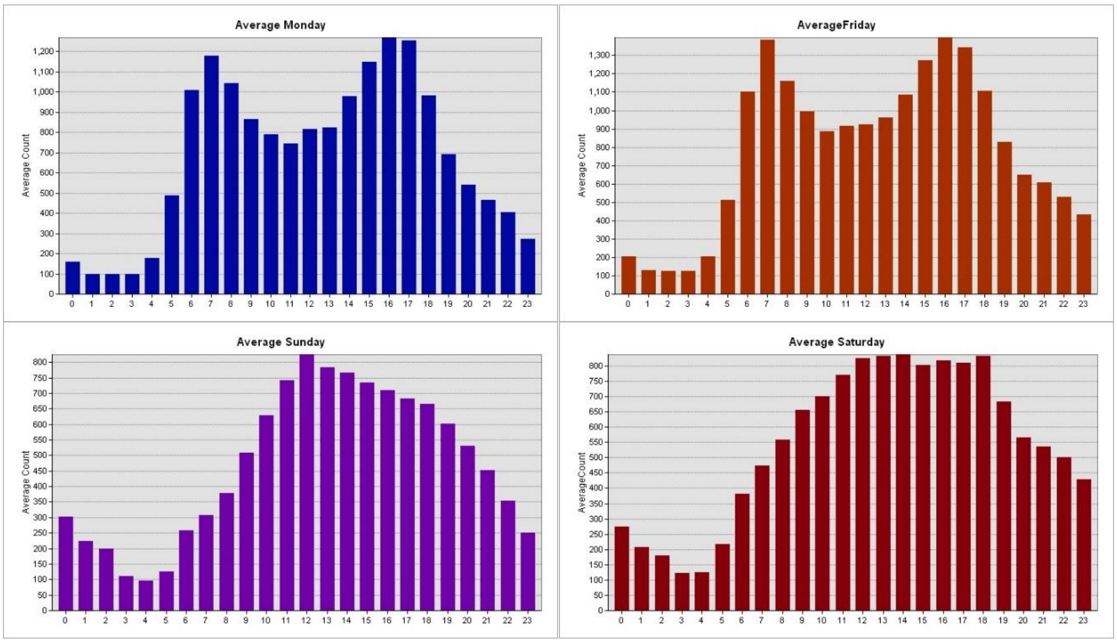

Figure S1. Annual average temporal distribution for particular days of the week as derived from Baltimore Metropolitan Council traffic data.

Tables

Table S1. A crosswalk between the Maryland and Baltimore governments' parcel and building data descriptions and the CBECS/RECS/MECS building descriptions.

**Parcel Description**	**CBES RECS MECS Description**
Apartments OTHER Building Per Square Foot	SINGLE-FAMILY DETACHED HOUSE
Apartments OTHER Building Per Unit	APARTMENT BUILDING WITH 2-4 UNITS
AUTO Auto Center	Retail other than mall
AUTO Auto Dealership Complete	Retail other than mall
AUTO Auto Showroom	Retail other than mall
AUTO Parking Structure	Other
AUTO Parking Structure - Split parcel	Other
AUTO Service Garage	Service
AUTO Service Garage -Split Parcel	Service
AUTO Service Mini Lube	Service
AUTO Service Station	Service
AUTO Service Storage Garage	Service
BANK Bank Branch	Office
BANK Bank Main	Office
BUILDING UNDER CONSTRUCTION 2008	Other
BURIAL Mortuary	Retail other than mall
CARE Day Care Center	Education
CARE Dental Clinic	Outpatient health care
CARE Dispensary	Office
CARE Group Home	Nursing
CARE Home for the Elderly	Nursing
CARE Hospital Convalescent	Inpatient health care
CARE Hospital General	Inpatient health care
CARE Nursing Home	Nursing
COMMERCIAL BUILDING Uncategorized	Office
Commercial OTHER Building Per Unit	Office
COMMUNITY Building Restroom	Public assembly
COMMUNITY Building Shower	Public assembly
COMMUNITY Church	Religious worship
COMMUNITY Library	Education
COMMUNITY Post Office Branch	Office
COMMUNITY Post Office Main	Office
COMMUNITY Rectory	Religious worship
DWEL Rental Dwelling	APARTMENT BUILDING WITH 2-4 UNITS
Exempt Commercial OTHER Building Per Square *	Other

Table S1. Continued.

**Parcel Description**	**CBES RECS MECS Description**
HOUSING Apartment(s)	APARTMENT BUILDING WITH 2-4 UNITS
HOUSING Condominium(s)	APARTMENT BUILDING WITH 2-4 UNITS
HOUSING Mobile Home(s)	MOBILE HOME
HOUSING Rental Townhouse Unit(s)	APARTMENT BUILDING WITH 2-4 UNITS
HOUSING Residence	SINGLE-FAMILY ATTACHED HOUSE
HOUSING Residence Multiple	APARTMENT BUILDING WITH 2-4 UNITS
HOUSING Residential Apartment Unit(s)	APARTMENT BUILDING WITH 2-4 UNITS
HOUSING Residential/Retail Mixed	APARTMENT BUILDING WITH 2-4 UNITS
INDUSTRY Engineering and Research	Miscellaneous
INDUSTRY Flex Space	Miscellaneous
INDUSTRY Industrial Heavy	Miscellaneous
INDUSTRY Industrial Shell	Miscellaneous
INDUSTRY Light Manufacturing	Miscellaneous
INDUSTRY Light Manufacturing - Split Parcel	Miscellaneous
INDUSTRY Loft	Miscellaneous
INDUSTRY Tank Farm	Miscellaneous
INSTITUTIONAL BUILDING Uncategorized	Public order and safety
MISC	Other
MISCELLANEOUS STRUCTURE	APARTMENT BUILDING WITH 2-4 UNITS
MISCELLANEOUS STRUCTURE Uncategorized	Other
OFFICE Building	Office
OFFICE Building Condominium	Office
OFFICE Building Medical	Office
OFFICE Building -Split Parcel	Office
OFFICE Veterinary Hospital	Office
PUBLIC Government Building	Public assembly
PUBLIC Government Building -Split parcel	Public assembly
PUBLIC Government Building -Split Parcel	Public assembly
REC Auditorium	Public assembly
REC Bowling Alley	Public assembly
REC City Club	Public assembly
REC Club House	Public assembly
REC Club House - Split Parcel	Public assembly
REC Country Club	Public assembly
REC Fraternal Building	Public assembly

Table S1. Continued.

**Parcel Description**	**CBES RECS MECS Description**
REC Gymnasium	Public assembly
REC Health Club	Public assembly
REC Skating Rink	Public assembly
REC Tennis Facility Indoor	Public assembly
REC Theater	Public assembly
RESIDENTIAL BUILDING Uncategorized	SINGLE-FAMILY ATTACHED HOUSE
Residential Condominium	APARTMENT BUILDING WITH 2-4 UNITS
RESTAURANT	Food service
RESTAURANT Fast Food	Food service
RESTAURANT Food Booth	Food service
RESTAURANT Tavern	Food service
SAFETY Armory	Public order and safety
SAFETY Fire Station	Public order and safety
SAFETY Fire Station Volunteer	Public order and safety
SAFETY Jail	Public order and safety
SCHOOL Building Classroom	Education
SCHOOL Building Computer Center	Education
SCHOOL Building Dormitory	Education
SCHOOL Building Fraternity House	Education
SCHOOL Building Laboratory	Education
SCHOOL Building Manual Arts	Education
SCHOOL Building Multi-Purpose School	Education
SCHOOL Elementary	Education
SCHOOL High	Education
STORE Beauty and/or Barber Shop	Retail other than mall
STORE Convenience	Retail other than mall
STORE Department	Retail other than mall
STORE Discount	Retail other than mall
STORE Discount -Split Parcel	Retail other than mall
STORE Laundromat	Retail other than mall
STORE Mall Enclosed	Enclosed mall
STORE Market	Retail other than mall
STORE Retail	Retail other than mall
STORE Retail Condominium	Retail other than mall
STORE Retail -Split Parcel	Retail other than mall
STORE Shopping Center Community	Enclosed mall
STORE Shopping Center Neighborhood	Enclosed mall
STORE Shopping Center Regional	Enclosed mall
STRY 1 1/2 Story No Basement	SINGLE-FAMILY DETACHED HOUSE

Table S1. Continued.

**Parcel Description**	**CBES RECS MECS Description**
STRY 1 1/2 Story With Basement	SINGLE-FAMILY DETACHED HOUSE
STRY 1 Story No Basement	SINGLE-FAMILY DETACHED HOUSE
STRY 1 Story With Basement	SINGLE-FAMILY DETACHED HOUSE
STRY 2 1/2 Story No Basement	SINGLE-FAMILY DETACHED HOUSE
STRY 2 1/2 Story With Basement	SINGLE-FAMILY DETACHED HOUSE
STRY 2 Story No Basement	SINGLE-FAMILY DETACHED HOUSE
STRY 2 Story With Basement	SINGLE-FAMILY DETACHED HOUSE
STRY 3 Story No Basement	SINGLE-FAMILY DETACHED HOUSE
STRY 3 Story With Basement	SINGLE-FAMILY DETACHED HOUSE
STRY 3 Story With Basement -Split Parcel	SINGLE-FAMILY DETACHED HOUSE
STRY 4 Story With Basement	SINGLE-FAMILY ATTACHED HOUSE
STRY Split Foyer	SINGLE-FAMILY ATTACHED HOUSE
STRY TH Center 1 1/2 Story No Basement	SINGLE-FAMILY ATTACHED HOUSE
STRY TH Center 1 1/2 Story With Basement	SINGLE-FAMILY ATTACHED HOUSE
STRY TH Center 1 Story No Basement	SINGLE-FAMILY ATTACHED HOUSE
STRY TH Center 1 Story With Basement	SINGLE-FAMILY ATTACHED HOUSE
STRY TH Center 2 1/2 Story No Basement	SINGLE-FAMILY ATTACHED HOUSE
STRY TH Center 2 1/2 Story With Basement	SINGLE-FAMILY ATTACHED HOUSE
STRY TH Center 2 Story No Basement	SINGLE-FAMILY ATTACHED HOUSE
STRY TH Center 2 Story With Basement	SINGLE-FAMILY ATTACHED HOUSE
STRY TH Center 3 Story No Basement	SINGLE-FAMILY ATTACHED HOUSE
STRY TH Center 3 Story With Basement	SINGLE-FAMILY ATTACHED HOUSE

Table S1. Continued.

**Parcel Description**	**CBES RECS MECS Description**
STRY TH Center 4 Story No Basement	SINGLE-FAMILY ATTACHED HOUSE
STRY TH Center 4 Story With Basement	SINGLE-FAMILY ATTACHED HOUSE
STRY TH End 1 1/2 Story With Basement	SINGLE-FAMILY ATTACHED HOUSE
STRY TH End 1 Story No Basement	SINGLE-FAMILY ATTACHED HOUSE
STRY TH End 1 Story With Basement	SINGLE-FAMILY ATTACHED HOUSE
STRY TH End 2 1/2 Story No Basement	SINGLE-FAMILY ATTACHED HOUSE
STRY TH End 2 1/2 Story With Basement	SINGLE-FAMILY ATTACHED HOUSE
STRY TH End 2 Story No Basement	SINGLE-FAMILY ATTACHED HOUSE
STRY TH End 2 Story With Basement	SINGLE-FAMILY ATTACHED HOUSE
STRY TH End 3 Story No Basement	SINGLE-FAMILY ATTACHED HOUSE
STRY TH End 3 Story With Basement	SINGLE-FAMILY ATTACHED HOUSE
STRY TH End 4 Story No Basement	SINGLE-FAMILY ATTACHED HOUSE
STRY TH End 4 Story With Basement	SINGLE-FAMILY ATTACHED HOUSE
TRANSPORT Passenger Terminal	Public assembly
TRAVEL Hotel	Lodging
TRAVEL Motel	Lodging
Unknown	APARTMENT BUILDING WITH 2-4 UNITS
WAREHOUSE	Nonrefrigerated warehouse
WAREHOUSE Cold Storage Facility	Refrigerated warehouse
WAREHOUSE Discount	Nonrefrigerated warehouse
WAREHOUSE Distribution	Nonrefrigerated warehouse
WAREHOUSE Mega	Nonrefrigerated warehouse
WAREHOUSE Mini Storage	Nonrefrigerated warehouse
WAREHOUSE Mini Storage Multi Story	Nonrefrigerated warehouse
WAREHOUSE Storage	Nonrefrigerated warehouse
WAREHOUSE Transit	Nonrefrigerated warehouse
X AUTO Car Wash	Service
X AUTO Dealership	Service
X AUTO Dealership Used Car Lot	Other
X AUTO Parking	Other

Table S1. Continued.

**Parcel Description**	**CBES RECS MECS Description**
X AUTO Parking Lot	Other
X AUTO Service	Service
X AUTO Service Station	Service
X AUTO Service Station w/Convenience Store	Service
X AUTO Service Storage Garage	Service
X BURIAL Funeral Home	Other
X CARE Nursing Home	Nursing
X COMMUNITY Church/Rectory/Mosque/Synagogue*	Religious worship
X COMMUNITY School	Education
X HOUSING Apartment	APARTMENT BUILDING WITH 2-4 UNITS
X HOUSING Apartment Garden	APARTMENT BUILDING WITH 2-4 UNITS
X INDUSTRY Manufacturing	Miscellaneous
X INDUSTRY Tank Farm	Miscellaneous
X INDUSTRY Truck Terminal	Transportation Equipment
X MISC Residence on Commercial/Industrial Z*	Other
X OFFICE Building	Office
X STORE Barber Shop or Hair Salon	Retail other than mall
X STORE Retail	Retail other than mall
X STORE Retail with Apartment Upstairs	Retail other than mall
X TELECOM Communication Tower	Other
X AUTO Car Wash	Service
X AUTO Dealership Used Car Lot	Service
X AUTO Junkyard	Other
X AUTO Parking	Other
X AUTO Parking Garage	Other
X AUTO Parking Lot	Service
X AUTO Service	Service
X AUTO Service Station	Service
X AUTO Service Station w/Convenience Store	Service
X AUTO Service Storage Garage	Service
X BANK Branch	Office
X BURIAL Cemetery	Other
X BURIAL Funeral Home Converted Dwelling	Retail other than mall
X CARE Ambulatory Assisted Living Facility	Nursing
X CARE Day Care Center	Education
X CARE Day Care Center Converted Dwelling	Education
X CARE Hospital	Inpatient health care

Table S1. Continued.

**Parcel Description**	**CBES RECS MECS Description**
X CARE Nursing Home	Nursing
X Commercial	Other
X COMMUNITY Church/Rectory/Mosque/Synagogue/*	Religious worship
X COMMUNITY Fraternity	Education
X COMMUNITY School	Education
X ELEC Baltimore Gas & Electric (OP)	Miscellaneous
X Exempt Commercial	Other
X HOUSING Apartment	APARTMENT BUILDING WITH 2-4 UNITS
X HOUSING Apartment Garden	APARTMENT BUILDING WITH 2-4 UNITS
X HOUSING Apartment High Rise	APARTMENT BUILDING WITH 2-4 UNITS
X HOUSING Apartment Mixed	APARTMENT BUILDING WITH 2-4 UNITS
X HOUSING Apartment Senior Unit	APARTMENT BUILDING WITH 5 OR MORE UNITS
X HOUSING Apartment Subsidized	APARTMENT BUILDING WITH 2-4 UNITS
X HOUSING Apartment Townhouse	APARTMENT BUILDING WITH 2-4 UNITS
X HOUSING Subsidized Housing Section 42 with*	SINGLE-FAMILY ATTACHED HOUSE
X HOUSING Trailer Park	MOBILE HOME
X Industrial	Miscellaneous
X INDUSTRY Manufacturing	Miscellaneous
X INDUSTRY Tank Farm	Miscellaneous
X MISC No Value Common Use Facility	Other
X MISC Residence on Commercial/Industrial Zo*	SINGLE-FAMILY DETACHED HOUSE
X OFFICE Building	Office
X OFFICE Building Condo	Office
X OFFICE Building Converted Dwelling	Office
X OFFICE Building Medical	Office
X OFFICE Building Veterinary	Office
X OTHER Yard Item such as Perimeter Fence	Other
X PIPE Columbia Gasoline Transmission (OP)	Miscellaneous
X PUBLIC County Property	Other
X PUBLIC Municipal Property	Other
X PUBLIC State Property	Other
X RAIL Conrail (NON-OP)	Other
X RAIL CSX Transportation (NON-OP)	Miscellaneous

Table S1. Continued.

Parcel Description	CBES RECS MECS Description
X RAIL CSX Transportation (OP)	Miscellaneous
X RAIL Norfolk & Southern (OP)	Miscellaneous
X REC Amusement Park	Public assembly
X REC Camp Ground	Public assembly
X REC Country Club Subject to Use Agreemnt	Public assembly
X REC Recreational Property	Public assembly
X REC Social Club	Public assembly
X Residential	SINGLE-FAMILY ATTACHED HOUSE
X RESTAURANT	Food service
X RESTAURANT Converted Dwelling	Food service
X RESTAURANT Fast Food	Food service
X SAFETY Armory	Public order and safety
X SAFETY Fire Station	Public order and safety
X SAFETY Jail or Correctional Facility	Public order and safety
X STEAM Baltimore Steam Company (OP)	Miscellaneous
X STORE Convenience Market	Retail other than mall
X STORE Laundromat	Retail other than mall
X STORE Liquor	Retail other than mall
X STORE Lumber Yard	Other
X STORE Retail	Retail other than mall
X STORE Retail with Apartment Upstairs	Retail other than mall
X STORE Shopping Center	Enclosed mall
X TELECOM Communication Tower	Other
X TELECOM Verizon Maryland (OP)	Other
X TRANSPORT Airport	Public assembly
X TRANSPORT Mass Transit Station	Public assembly
X TRAVEL Hotel	Lodging
X TRAVEL Motel	Lodging
X WAREHOUSE	Nonrefrigerated warehouse
X WAREHOUSE Loft/Research & Development/Flex	Nonrefrigerated warehouse
X WAREHOUSE Machinery/Equipment Shed	Nonrefrigerated warehouse

Table S2. Crosswalk between CBECS building types and DOE2/eQUEST building types.

**CBECS building code**	**CBECS building type**	**eQUEST building type**
2	Office	Office Bldg, mid-rise
5	Nonrefrigerated warehouse	Retail, Warehouse Sales
7	Public order and safety	Unknown, Custom or Mixed Use
8	Outpatient health care	Health, Medical Clinic/Prof.Bldg (outpatient)
11	Refrigerated warehouse	Retail, Warehouse Sales
12	Religious worship	Religious worship
13	Public assembly	Unknown, Custom or Mixed Use
14	Education	School, Secondary (High School)
15	Food service	Restaurant, Full Service (full menu)
16	Inpatient health care	Health, Hospital (inpatient)
17	Nursing	Health, Long-term Care (Nursing Home)
18	Lodging	Lodging, High-Rise Hotel
24	Enclosed mall	Retail, Department Store
25	Retail other than mall	Retail, Stand-Alone Structure
26	Service	Retail, Service Station
91	Other	Unknown, Custom or Mixed Use

Table S3. Crosswalk between RECS building types and DOE2/eQUEST building types.

**RECS building code**	**RECS building type**	**eQUEST building type**
1	MOBILE HOME	Multifamily, Low-Rise (exterior entries)
2	SINGLE-FAMILY DETACHED HOUSE	Multifamily, Low-Rise (exterior entries)
3	SINGLE-FAMILY ATTACHED HOUSE	Multifamily, Low-Rise (exterior entries)
4	APARTMENT BUILDING WITH 2-4 UNITS	Multifamily, Low-Rise (exterior entries)
5	APARTMENT BUILDING WITH 5 OR MORE UNITS	Multifamily, Mid-Rise (interior entries)

References

1. Gurney KR, Liang J, Patarasuk R, Song Y, Huang J, Roest GS. The Vulcan Version 3.0 High-Resolution Fossil Fuel CO_2 Emissions for the United States. Earth Syst Sci Data Discuss. 2019;
2. Maryland Department of Planning. Download GIS Files [Internet]. [cited 2020 Jan 17]. Available from: https://planning.maryland.gov/Pages/OurProducts/downloadFiles.aspx
3. Baltimore City Government. Building Footprint - Shape | Open Baltimore | City of Baltimore Open Data Catalog [Internet]. [cited 2020 Jan 17]. Available from: https://data.baltimorecity.gov/Geographic/Building-Footprint-Shape/deus-s85f
4. Huang J, Akbari H, Rainer L, Ritschard R. 481 Prototypical Commercial Buildings for 20 Urban Market Areas [Internet]. Berkeley, CA; 1991 [cited 2020 Jan 8]. Available from: https://gundog.lbl.gov/dirpubs/29798.pdf
5. Gurney KR, Razlivanov I, Song Y, Zhou Y, Benes B, Abdul-Massih M. Quantification of fossil fuel CO_2 emissions on the building/street scale for a large U.S. City. Environ Sci Technol. 2012;46(21):12194–202.
6. Patarasuk R, Gurney KR, O’Keeffe D, Song Y, Huang J, Rao P, et al. Urban high-resolution fossil fuel CO_2 emissions quantification and exploration of emission drivers for potential policy applications. Urban Ecosyst. 2016;19(3):1013–39.
7. Gurney KR, Patarasuk R, Liang J, Song Y, O'Keeffe D, Rao P, et al. The Hestia fossil fuel CO_2 emissions data product for the Los Angeles megacity (Hestia-LA). Earth Syst Sci Data [Internet]. 2019 Aug 26 [cited 2020 Jan 29];11(3):1309–35. Available from: https://www.earth-syst-sci-data.net/11/1309/2019/

8. Baltimore Metropolitan Council. Traffic Count Database [Internet]. [cited 2020 Jan 17]. Available from: https://www.baltometro.org/transportation/data-maps/traffic-count-database
